# Severe Vaginal Bleeding in a Case of Renal Cell Carcinoma

**DOI:** 10.1155/2019/2174051

**Published:** 2019-05-27

**Authors:** Ryan Machiele, Taylor Renbarger, Bret Guidry

**Affiliations:** ^1^Campbell University School of Osteopathic Medicine in Lillington, North Carolina, USA; ^2^Cape Fear Valley OBGYN Residency in Fayetteville, North Carolina, USA

## Abstract

Renal Cell Carcinoma (RCC) accounts for approximately 2-3% of all adult cancers and carries the highest mortality of the genitourinary cancers. Metastatic disease is seen in approximately 16% of cases and when present represents an advanced status. Metastasis of RCC to the vagina has rarely been cited in literature and when present can mimic primary vaginal cancer in clinical presentation and symptoms. Biopsy is performed to delineate the etiology and, in the presence of clear cells and certain immunohistochemistry markers, RCC needs to be included in the differential diagnosis. Treatment protocols are limited due to the rarity of the condition, with retrospective and comparative studies alongside cervical cancer treatment protocols serving as the basis. Herein, we describe a unique case of profuse vaginal bleeding secondary to vaginal metastases of RCC and discuss the relevant aspects of diagnosis and treatment.

## 1. Introduction

We present an unusual case of profuse vaginal bleeding due to metastases of Renal Cell Carcinoma (RCC). RCC incidence varies with location, with North American men in their 60's-80's at greatest risk. In the US, approximately 74,000 new cases are diagnosed each year, with 15,000 deaths attributed to this condition annually [1]. Risk factors include end-stage renal disease (ESRD), acquired cystic kidney disease (ACKD), smoking, hypertension, occupational exposure and genetic factors, among others. RCC comprises approximately 2-3% of all adult cancers and has the highest mortality of all genitourinary cancers [2, 3]. It has an indolent course in many patients: one third of patients already have advanced disease that is locally invasive or metastatic at presentation [3]. When symptomatic, the classical triad of a palpable flank mass, flank pain, and hematuria occurs approximately 9% of the time and signifies a more advanced disease [4]. The presence of anemia in advanced disease is seen in approximately 88% of cases and often precedes other symptoms by several months. Metastatic disease occurs in 16% of cases, with the most common locations being the lung, lymph nodes, bone, liver, and brain [2]. Despite the prevalence of metastatic RCC, metastasis to the vagina is an exceedingly rare phenomenon with fewer than 100 reported cases [5]. We present herein a case of a 60-year-old female who presented with severe vaginal bleeding found to be caused by vaginal metastases of RCC.

## 2. Case Report

A 60-year-old female presented to the Emergency Department (ED) with a complaint of left abdominal pain for the preceding two months. She stated that the pain was moderate in intensity and radiated to the midline with no aggravating or alleviating factors. She also complained of vaginal bleeding, intermittent dysuria, and generalized weakness. She denied fever, night sweats, and headache. Ultrasound done at the time revealed a right nephrectomy and an enlarged left kidney with a multifocal left renal mass thought to represent a neoplasm. Urology and oncology were consulted at that time.

This patient's surgical history included a c-section and a tubal ligation. The full gynecologic history timeline was somewhat limited as this patient had recently moved to the area after receiving care out-of-state with limited records. Past medical history included a diagnosis of right-sided RCC with subsequent right radical nephrectomy 6 years prior and diagnosis of a plasma cell neoplasm 6 months prior. Most recent history included a diagnosis of anemia made in the past month by the patient's nephrologist. CT scan imaging done at this visit revealed a nearly complete replacement of the left kidney by abnormal mass-like enhancement with internal neovascularity, thought to represent a renal neoplasm. The patient was instructed to follow up for a confirmatory renal biopsy and failed to do so, with three weeks passing between this time and her arrival to the ED.

**Figure 1:**
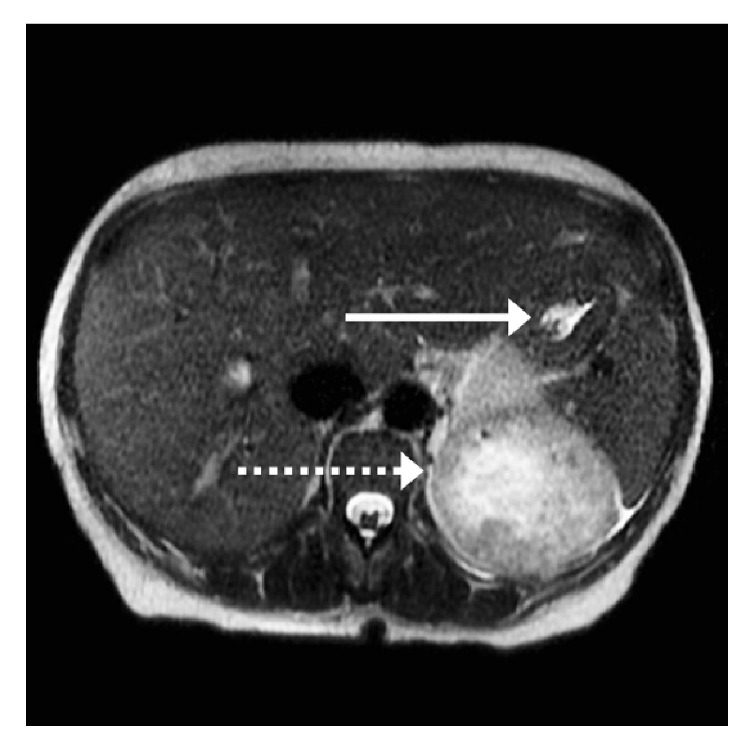
Axial & coronal view, CT of abdomen without contrast demonstrating multifocal left renal malignancy (dashed arrows) with metastasis to vagina as well as left hepatic lobe (solid arrows).

**Figure 2:**
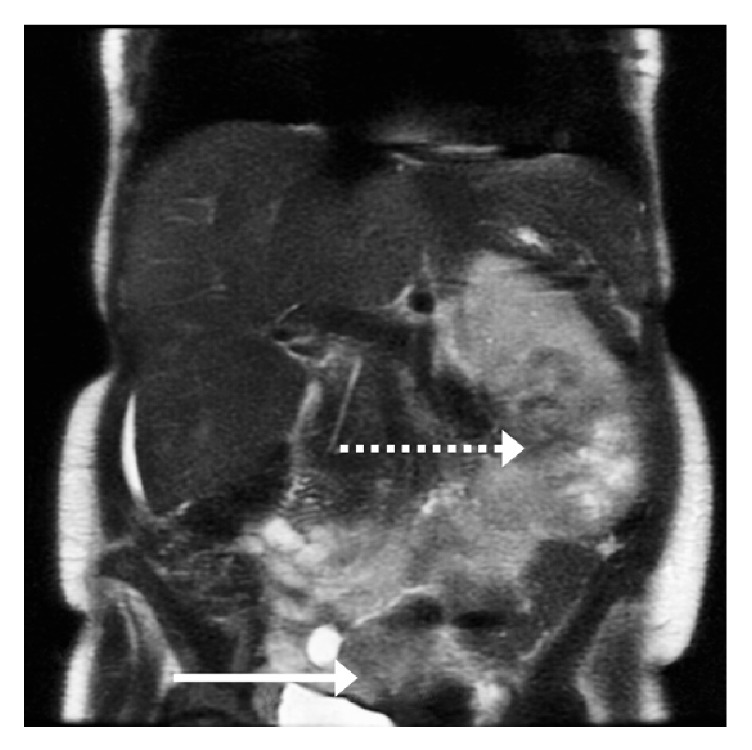
Axial & coronal view, CT of abdomen without contrast demonstrating multifocal left renal malignancy (dashed arrows) with metastasis to vagina as well as left hepatic lobe (solid arrows).

Upon presentation to the ED, laboratory data revealed a hemoglobin of 6.9 g/dl and a mean corpuscular volume of 79.2 femtoliters consistent with microcytic anemia. Fecal occult blood was negative and urinalysis was positive for 6+ red blood cells. CT imaging findings were consistent with a multifocal left renal malignancy with metastasis to vagina as well as left hepatic lobe (Figures 1 and 2). Pelvic examination revealed a significant amount of blood in the vaginal vault with active bleeding noted. The obstetric and gynecological team was consulted, noting an active life-threatening vaginal hemorrhage.

**Figure 3:**
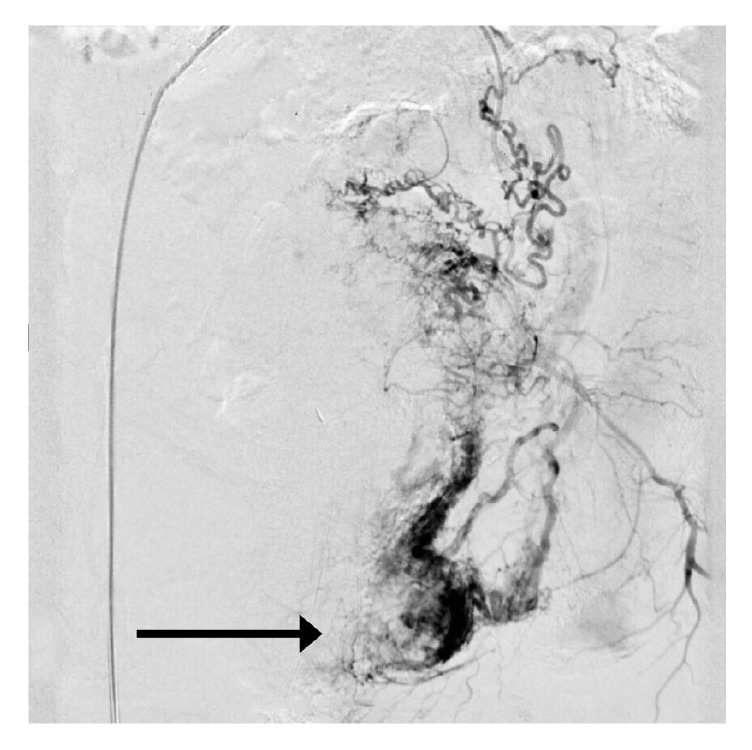
Pelvic arteriogram demonstrating a large hypervascular blush with active extravasation (arrow) along the left side of the vagina consistent with metastatic lesion.

Informed consent was obtained, and the patient was taken to the operating room for exam under anesthesia, vaginal biopsy and vaginal packing. Exam using sidewall retractors revealed active bleeding from a necrotic mass which was not amenable to suture ligation or electrocautery. The mass was located on the left vaginal sidewall, lateral, and posterior to the hymenal ring, measuring approximately 3.5 x 1 cm in size. The mass was biopsied, and the vagina was packed with monsel-soaked Kerlix. While still intubated the patient was taken to the interventional radiology suite for artery embolization. In this phase of treatment, embolization was performed of the left vaginal artery, pudendal artery, and collateral vessels of the obturator artery using 300-500 *μ*m Embospheres and bilateral anterior divisions of the internal iliac arteries using Gelfoam. A pelvic arteriogram was performed and showed a large hypervascular mass with active extravasation of the left vagina consistent with metastatic disease (Figure 3).

**Figure 4:**
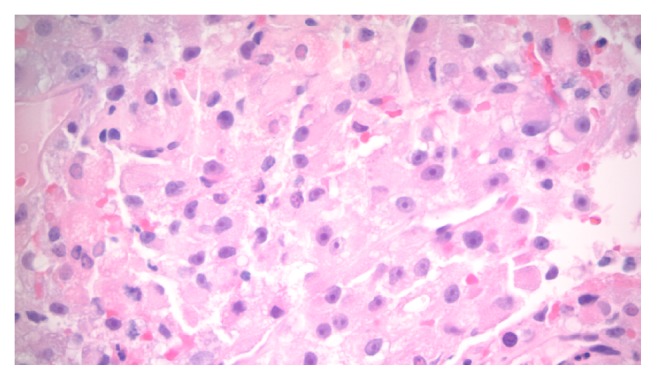
Vaginal mass biopsy: Hematoxylin and Eosin stain at 40X magnification.

**Figure 5:**
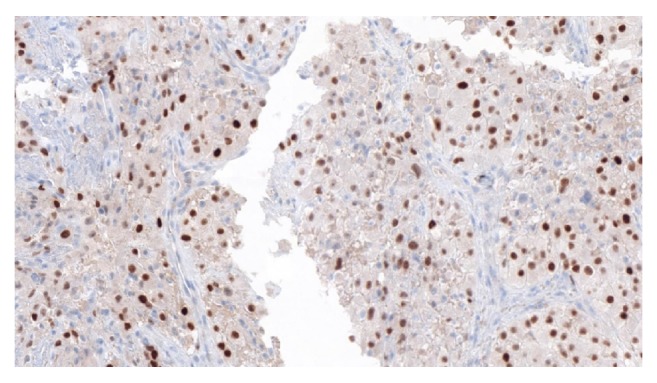
Vaginal mass biopsy: demonstrating positive reactivity for immunohistochemical PAX8 Stain, consistent with RCC.

**Figure 6:**
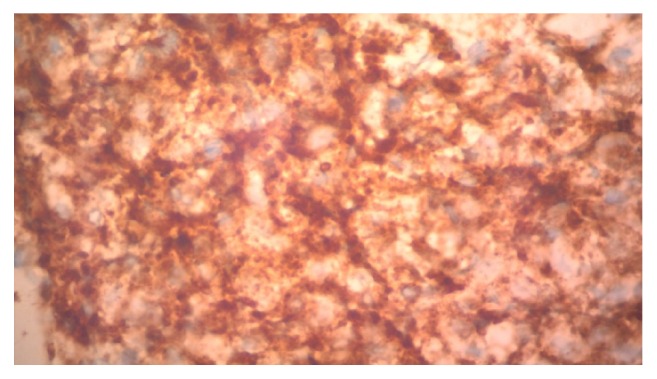
Vaginal mass biopsy: demonstrating positive reactivity for immunohistochemical CD10 Stain, consistent with RCC.

Two formalin-fixed biopsy specimens measuring 0.45 x 0.45 x 0.15 cm and 0.5 x 0.4 x 0.2 cm in size were sent to pathology for consideration (Figure 4). The two specimens were found to be positive for vimentin, CK8/18, AE1/3, CD10, and PAX8 (Figures 5 and 6). They were negative for CK7, EMA, CD117, and MART-1. These findings are consistent with a diagnosis of metastatic RCC.

## 3. Discussion

This case illustrates the importance of maintaining a broad differential and applying past medical history when investigating the etiology of abnormal uterine bleeding in the postmenopausal female. The range of etiologies contributing to abnormal uterine bleeding after menopause is expansive. A study of 1220 female patients presenting with abnormal uterine bleeding recorded the following diagnoses within the post-menopausal stratum (454): 37.7% due to polyps, 30.8% due to vaginal/endometrial atrophy, 14.5% due to abnormal proliferative/secretory function, 6.6% due to carcinoma, 6.2% due to fibroids, 2% due to hyperplasia without atypia, and 0.2% due to atypical hyperplasia [6].

Primary vaginal cancer makes up approximately 1-2 % of all gynecological malignancies [7]. Risk of primary vaginal cancer increases with age, with peak incidence seen in the 6th and 7th decades [8]. Therefore, as the postmenopausal female ages, vigilance surrounding abnormal bleeding becomes increasingly critical. Histology of primary vaginal tumors shows a strong tendency toward squamous cell carcinoma, with clear cell adenocarcinoma making up a smaller proportion. Persistence of HPV is the most common cause of squamous cell carcinoma. Intrauterine exposure to diethylstilbestrol is the most commonly associated cause of clear cell adenocarcinoma, although it can occur without [9]. The majority of vaginal tumors are not primary malignancies but are metastases of systemic cancer, most often a result of direct extension of endometrial or cervical cancer or hematologic spread of breast or ovarian cancer [10, 11]. The frequency of RCC metastatic to the vagina has been described fewer than 100 times in the literature, reflecting a low incidence of disease with the mechanism of spread not entirely understood. It is postulated that retrograde venous flow via the ovarian vein facilitates seeding of the vaginal mucosa from the kidneys, making left sided RCC more likely to result in vaginal metastasis due to aberrant flow between the left ovarian vein and the left renal vein [12]. Radiological studies have observed flux in venous flow in patients with RCC accompanied by vaginal metastasis [13]. The individual under consideration presented with vaginal cancer in the setting of left-sided RCC, further adding to the legitimacy of this unique mechanism of spread.

Biopsy is used as the definitive method of diagnosis; however histopathologic analysis of the tumor is complicated by the considerable morphological overlap between clear cell renal cell carcinoma (CCRCC) and vaginal clear cell carcinoma. Both processes may present with solid, lobular architecture with similar prevalence of confluent glands and acini with intraluminal blood. A clinicopathologic analysis of 17 cases of CCRCC metastatic to the gynecologic tract recognized alveolar, acinar, and nested patterns as key differentiating features with significant association with CCRCC. This study found that while many cases of gynecologic clear cell carcinoma show predominantly papillary architecture and hobnail cells, these findings were absent in all 17 cases of CCRCC metastatic to the gynecologic tract and therefore add confidence to a differential diagnosis. Because of the overlap in morphology, immunohistochemistry is critical for diagnosis. This study found CA-IX, CD10, PAX-8, and RCC antigen reactivity strongly associated with CCRCC but again found overlap with gynecologic clear cell adenocarcinoma also expressing CD10 and PAX-8. Ultimately, CA-IX and RCC antigen have the strongest diagnostic value for CCRCC as they have no expression in gynecologic clear cell adenocarcinoma [14]. Due to the significant overlap in clinical presentation, histology, and immunohistochemistry markers, the need to correlate findings with past medical history becomes vital. The individual under consideration in this case was positive for CD10 and PAX-8, but with a past medical history including RCC, RCC metastasis becomes more likely in the absence of other risk factors for vaginal cancer.

Due to the rarity of vaginal cancers, treatment is based on retrospective and comparative studies alone, resulting in a variety of treatment plans implemented [7]. The acute presentation of active, profuse vaginal bleeding in this patient necessitated immediate surgical intervention as opposed to biopsy alone. Preoperative blood loss alone was estimated to be 800 mL, and intraoperative control of bleeding could not be achieved with standard surgical techniques such as suture ligation and electrocautery. This led to the decision to pursue control of bleeding via uterine artery embolization. Percutaneous transcatheter embolization of uterine arteries is commonly applied in the setting of fibroids and postpartum hemorrhage but has not been used in the setting of metastatic RCC. In this case, it proved to be effective in controlling bleeding due to metastatic vaginal malignancy. Prior cases of vaginal cancers have been treated with surgical resection, but this is often complicated by the close proximity of the bladder and rectum. Data regarding acute treatment of vaginal bleeding in vaginal carcinoma is limited. Treatment is often extrapolated from cervical cancer data, and chemoradiation is often added [15].

## 4. Conclusion

Primary vaginal cancer accounts for 1-2% of vaginal malignancies, with metastasis more commonly implicated. Regardless of etiology, vaginal cancer presents with bleeding that can be minimal to profuse. Due to the similarities in clinical presentation between primary vaginal cancer and metastasis, diagnosis depends on biopsy of the lesion with immunohistochemistry. The presence of clear cells with chemistry markers PAX-8, CA-IX, CD10 and RCC antigen is consistent with RCC, a highly malignant cancer associated with the highest mortality of the genitourinary cancers. Metastasis of RCC to the vagina is rare, but can result devastating if not promptly diagnosed and treated. This case demonstrates a novel and effective application of percutaneous transcatheter artery embolization in the setting of uncontrollable vaginal bleeding caused by RCC vaginal metastasis. The pathophysiology behind metastasis of RCC to the vagina is not well understood and further evaluation of this condition needs to be investigated.
